# MAT cross-reactions or vaccine cross-protection: retrospective study of 863 leptospirosis canine cases

**DOI:** 10.1016/j.heliyon.2018.e00869

**Published:** 2018-11-02

**Authors:** Geneviève André-Fontaine, Laetitia Triger

**Affiliations:** Laboratoire de Bactériologie Médicale et Moléculaire des Leptospires, École Nationale Vétérinaire, ONIRIS, Route de Gachet, CS 40706, 44307 Nantes Cedex 03, France

**Keywords:** Veterinary science, Veterinary medicine

## Abstract

Dogs are naturally exposed to numerous pathogenic serogroups. Leptospirosis vaccines are claimed to afford a clinical protection restricted to the serogroups of which they are composed.

**Objectives:**

Dogs exhibiting liver and kidney injury were suspected of having leptospirosis. The purpose of this study was to compare the microscopic agglutination test (MAT) results in naive and vaccinated dogs experiencing leptospirosis outcomes. Only MAT-positive animals were included in the study.

**Methods:**

Over five years, 3 512 dogs were suspected of having leptospirosis. For each case, biochemical parameter results were recorded. Leptospirosis involvement was investigated by MAT performed against 6 major serogroups (Icterohaemorrhagiae, Canicola, Australis, Autumnalis, Grippotyphosa and Sejroë). MAT-positive results confirmed leptospirosis cases in 147 naïve dogs and in 580 fully vaccinated dogs. Serological titres of agglutinating antibodies were related to the severity of liver and kidney failure.

**Results:**

The most prevalent outcome of leptospirosis in unvaccinated dogs was liver failure (57.8%) compared to 51.7% for kidney disease, but the most severe onset (90.8%) was found among the cases of acute kidney injury compared to the severe (42.3%) hepatitis cases. In dogs vaccinated by bivalent Icterohaemorrhagiae and Canicola bacterins, hepatitis decreased from 57.8 to 46.5% and acute kidney injury from 51.7 to 21.6%. The decrease was shown in leptospirosis cases induced by field strains belonging to the six most prevalent serogroups, including the 4 serogroups heterologous to the vaccine.

**Conclusion:**

Common vaccination was efficient in decreasing hepatitis and kidney failure induced by field *Leptospira* spp infection regardless of the MAT-prominent serogroup and limited the disease severity in the remaining cases.

## 1 Introduction

Acute leptospirosis with fatal outcome is commonly recorded in dogs, which are highly receptive to this type of infection [1]. More than 8 pathogenic serogroups have been detected worldwide in canines [2, 3, 4, 5]. The agglutinating antibodies raised against these pathogenic serogroups were shown by the serological microscopic agglutination test (MAT) from the tenth day after disease onset. MAT has been widely used to confirm leptospirosis cases [2, 6], indicating the most infectious serogroups. Formerly, canine vaccination was performed worldwide by bivalent whole cell vaccines, including the two major serogroups, Icterohaemorrhagiae (IH) and Canicola (CAN). The bacterins trigger agglutinating antibodies, which afford clinical protection. However, this protection is believed to be restricted to the composing serogroups of the vaccine [3]. The vaccine strategy to extend the protection against other pathogenic serogroups without any safety impairment was to add the most relevant among the approximately 25 available pathogenic serogroups [7, 8, 9] to be closer to the epidemiological status [10]. The goal of this study was to estimate the clinical protection afforded by vaccination in the field.

To avoid misdiagnosis, this retrospective study took place exclusively when Icterohaemorrhagiae and Canicola bivalent vaccines were only sold in France and when the MAT was the single tool for confirming leptospirosis cases [2, 11, 12]. Onset of leptospirosis was clinically suspected in 3 512 pets either naive (nV) or vaccinated with these bacterins. Clinical, biological and epidemiological data were recorded, and MAT was performed with each of the sera withdrawn from leptospirosis suspected dogs.

The study was only focused on dogs suspected of having leptospirosis due to exhibiting acute hepatitis and/or nephritis. The first step was to determine the liver and kidney injury patterns of each of the 6 major serogroups based on the MAT results of unvaccinated dogs. Therefore, comparison of the cases recorded in vaccinates and naïve cases allowed a global estimation of the serological and clinical impacts triggered by the usual whole cell bacterins.

## 2 Materials and methods

### 2.1 Data collection

A form was distributed among veterinary practitioners to collect clinical data of pets suspected of having leptospirosis.

The template, necessary to confirm or reject a leptospirosis case by MAT results, was prepared by the laboratory:

• Delay of sampling since the onset of the disease
• Delay of sampling since the last leptospirosis vaccination (primo vaccination or boost, without any record of the vaccine company).

### 2.2 Symptom criteria collection

• Liver injury (L): results of APL, ALT and biliary acids, icterus…
• Acute kidney injury (AKI): blood urea (BUN) and creatinine…
• Gastroenteritis, vomiting.
• Other symptoms relevant to leptospirosis, including bleeding, pulmonary distress or reproductive disorders.

Three grades of liver injury were defined by comparison with normal values (Table 1) [13, 14]. The third grade was estimated as severe liver injury (SL). Grades of acute kidney injury (AKI) were designated according to the International Renal Interest Society recommendations [15]. Grades III to V were representative of severe AKI (SK). Symptoms were independently recorded for each dog. Therefore, each syndrome had to be independently studied.

**Table 1:** Selected biochemical parameters used in grading liver and kidney injuries: III: severe liver damage, III–V: severe AKI.

Liver						Kidney			
Grade	Biliary acid μmol/L	ALAT UI	PAL UI	Jaundice		Grade	Creat mg/L	BUN g/L	
I	30–50	100–120	200–400	.+/−		I-II	<30	0.5–0.9	
II	50–70	120–200	400–600	.+	*>Alpha 2, Beta, coloured urine*	III–IV	30–40	1–1.50	
III	>70	>200	>600	.+++	*Ascitis*	IV–V	>40	>1.50	*Proteinuria, cylindrical cells*

### 2.3 MAT

As previously described [4, 5], the dogs sampled in France showed MAT-positive results to 6 major serogroups, the bacterin serogroups (I**cterohaemorrhagiae** and Canicola), and Australis (AUS), Autumnalis (AUT), Grippotyphosa (GRIP) and Sejroë (SEJ). A panel of 16 serovars (reference strains and local isolates), routinely used for these 6 serogroups, were used in this study.

A titre of 1:40 was considered positive. Nevertheless, in vaccinated animals, Icterohaemorrhagiae and Canicola reciprocal titres from 40 to 320 were considered as post-vaccination titres. The prominent serogroup was designed by the serovar exhibiting the highest titre, regardless of the positive serovar.

### 2.4 Case definition

The completion of the data sheet was necessary for any case confirmation.

A leptospirosis case was first excluded if the data related to the 5 selected biochemical and epidemiological factors were not relevant to clinical leptospirosis, and in a second step if MAT results were negative. Confirmation was serologically assessed by positive MAT results according to the algorithm previously defined [16], which, briefly, took into account:

(1) Vaccination status (vaccinated for leptospirosis or not);
(2) Time lapse between the vaccination and the sampling (less or more than 6 months prior);
(3) Delay between the sampling time and the onset of the first clinical outcome (less or more than 10 days prior).
(4) MAT Icterohaemorrhagiae and Canicola reciprocal titres ≥40 in unvaccinated and ≥320 in vaccinated,).
(5) MAT titres for other serogroups ≥40.

### 2.5 Statistical analysis

The data forms and MAT results were stored by Access 2000 software.

The results were treated by Pearson's chi-squared tests. Results were significant for P ≤ 0.05. Confidence intervals were expressed for P = 0.05 (CI 95% = x_1_-x_2_).

## 3 Results

### 3.1 Canine population suspected of having leptospirosis

**Fig. 1:**
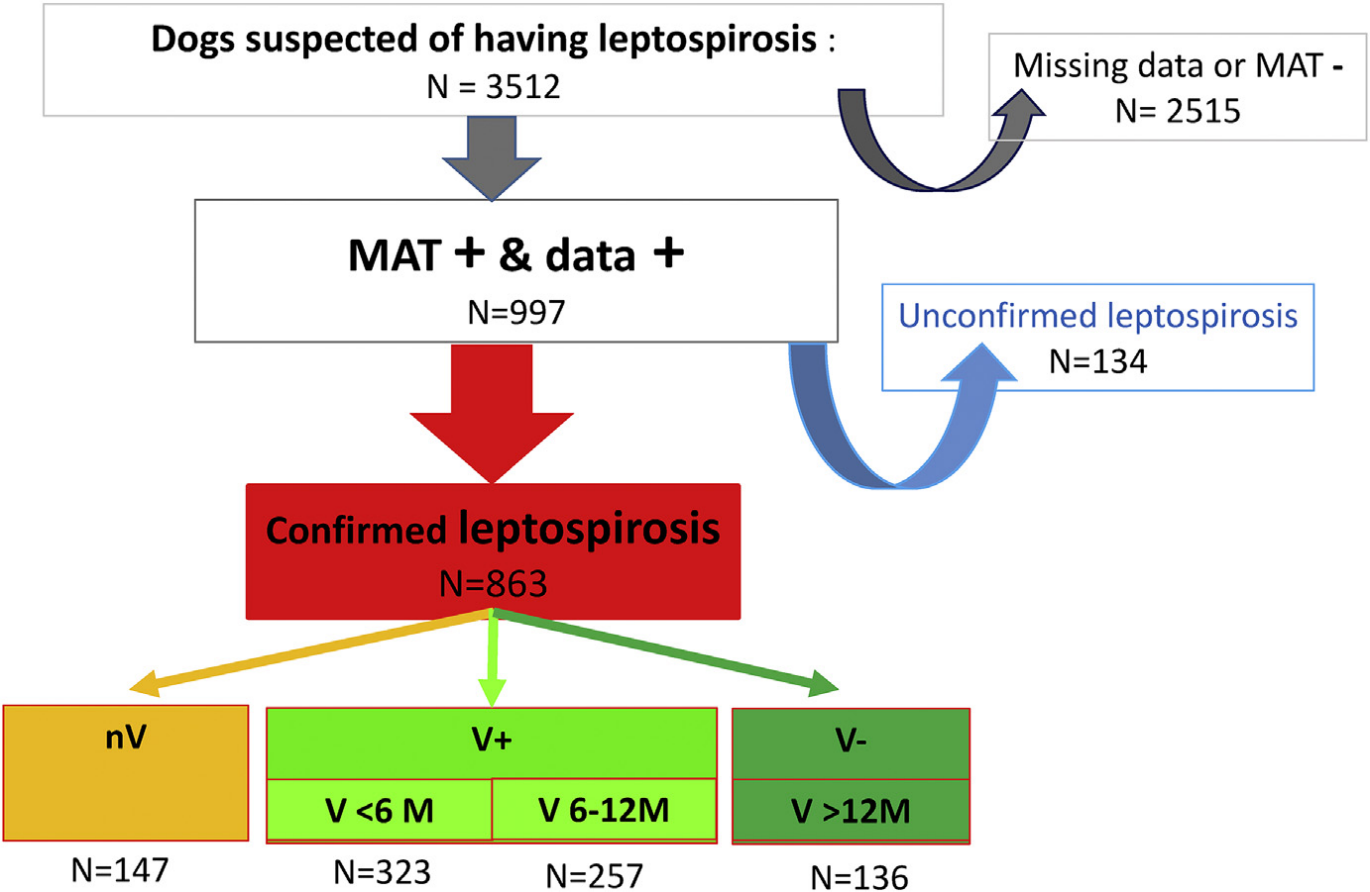
Studied population. nV: naive, V+: valid vaccination, V−: last vaccination >12 months.

Over the 5 years of the study, canine sera of 3 512 clinically suspected dogs were sent to the laboratory for MAT confirmation. Only 997 dogs suspected of having leptospirosis were determined to be suitable by combining fully completed forms and MAT-positive results (Fig. 1). From the 997 MAT-positive dogs, 863 leptospirosis cases were MAT confirmed according to the decision algorithm. Among the 863 confirmed cases, a group of 147 dogs were never vaccinated (nV). Among the 716 “vaccinated” dogs, 580 were valuably vaccinated (V+) according to the Pharmacopoeia recommendations (a valid vaccination is performed during the past year). The remaining group of 136 dogs (V−) were vaccinated more than 1 year prior and therefore could be considered neither naïve nor vaccinated at the onset of the disease.

### 3.2 MAT results

In MAT, a suspected dog belonging to the group of unvaccinated animals exhibited agglutinating antibodies to a mean of 2.36 different serogroups (347 MAT-positive results against the 6 most reactive serogroups). Sera samples provided by 580 vaccinates exhibited 1 506 positive results (mean = 2.60). However, when positive results for Icterohaemorrhagiae and Canicola were retrieved because they could be attributed to the previous vaccination performed in these animals, this mean dropped to 1.09.

The seroprevalence of each serogroup was estimated by using the threshold dilution of 1:40, but has to be evaluated according to these numerous responses.

### 3.2.1 Unvaccinated dogs

**Fig. 2:**
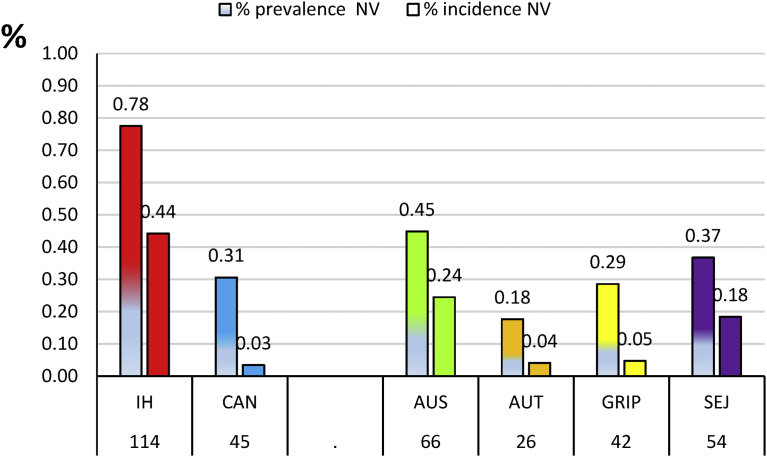
Prevalence (MAT+) and incidence (high titres) in percent of unvaccinated dogs (N = 147). IH: Icterohaemorrhagiae; CAN: Canicola; AUS: Australis; AUT: Autumnalis; GRIP: Grippotyphosa; SEJ: Sejroë; nV: not vaccinated dogs.

In the group of unvaccinated dogs, the highest seroprevalence of 77.6% (CI 95% = 70.9–84.3) was shown by Icterohaemorrhagiae (Fig. 2), followed by Australis 44.9% (CI 95% = 6.9–52.9), Sejroë 36.7% (CI 95% = 28.9–44.5), Canicola 30.6% (CI 95% = 23.2–38.1), Grippotyphosa 28.6% (CI 95% = 21.3–35.9) and Autumnalis 17.7% (CI 95% = 11.6–23.9).

The natural incidence of each serogroup could be roughly estimated by the highest reciprocal titres (≥320). The highest seroincidence (Fig. 2) among the 147 positive naive dogs was shown for the serogroup Icterohaemorrhagiae 44.2% (CI 95% = 26.2–52.2), followed by Australis 24.5% (CI 95% = 17.5–31.5), Sejroë 18.4% (CI 95% = 12.1–24.7). The 3 other serogroups were mildly seroreactive: Grippotyphosa incidence was 4.8% (CI 95% = 1.4–8.3), Autumnalis incidence was 4.1% (CI 95% = 0.9–6.7) and the lowest incidence was Canicola, which was 3.4% (CI 95% = 0.5–6.3).

Icterohaemorrhagiae infection was the most frequent compared to Australis (P = 4.10^−3^), to Sejroë (P = 8.10^−5^), Grippotyphosa (P = 1.10^−11^), Autumnalis (P = 3.10^−12^) and Canicola (P = 8.10^−13^).

### 3.2.2 Vaccinated dogs

In the group of vaccinated dogs, the Icterohaemorrhagiae and Canicola seroprevalences must be considered “apparent” prevalences as they included the serological consequences of previous vaccination. These seroprevalences were 88.6% (CI 95% = 86.8–91.2) for Icterohaemorrhagiae and 61.89% (CI 95% = 57.9–65.9) for Canicola. The 4 other serogroup seroprevalences were 35.9% for Sejroë (CI 95% = 32.0–39.8), 30.0% for Australis (CI 95% = 26.3–33.7), 22.9% for Grippotyphosa (CI 95% = 19.5–26.3) and 20.3% for Autumnalis (CI 95% = 17.0–23.6).

Similarly, incidence estimated by reciprocal high titres had to be studied separately from the other serogroups for Icterohaemorrhagiae and Canicola (≥320), which could be related to the previous *Leptospira* vaccination.

#### 3.2.2.1 *Icterohaemorrhagiae* and *Canicola*

**Fig. 3:**
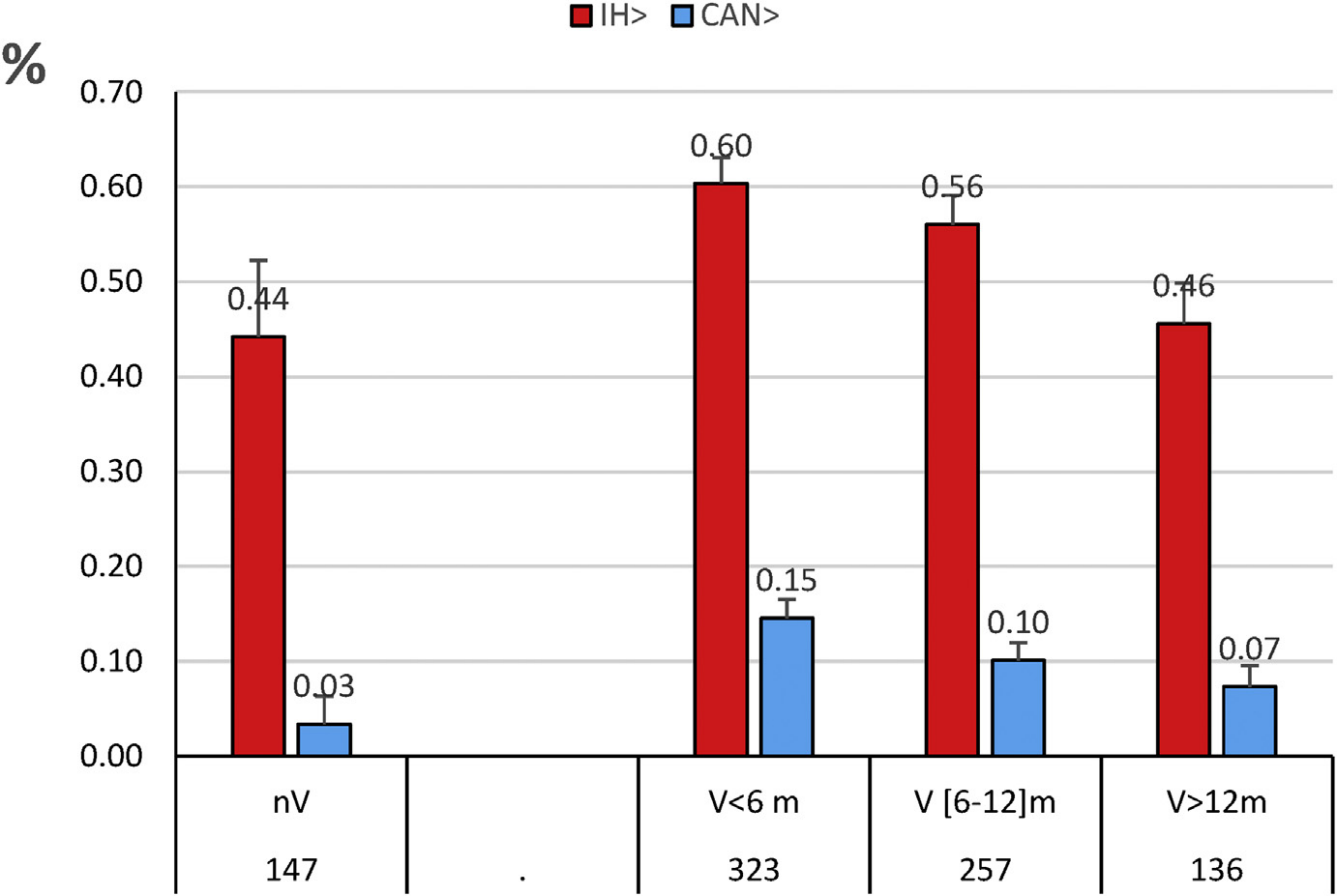
High titres against Icterohaemorrhagiae (IH) and Canicola (CAN) in 863 leptospirosis cases. In percent of each group of dogs. nV: not vaccinated; V < 6 m: last vaccination < six months; V [6 -12] m: last vaccination > six months and < 12 months; V > 12 m: last vaccination >12 months

High titres for Icterohaemorrhagiae and Canicola were found in 58.4% and 12.6% of the dogs, respectively. However, the titres against Icterohaemorrhagiae and Canicola decreased with the time elapsed since the last vaccination (Fig. 3). They decreased from 60% for Icterohaemorrhagiae and 15% against Canicola when the previous vaccination was performed during the previous 6 months to 45.6% and 7.4% in the group of 136 dogs whose vaccination was not valid (V-) (Fig. 3). This decrease of MAT results was significant for both Icterohaemorrhagiae and Canicola (P = 0.040 and P = 0.022) when the last vaccination was performed more than 1 year ago.

#### 3.2.2.2 Other serogroups

**Fig. 4:**
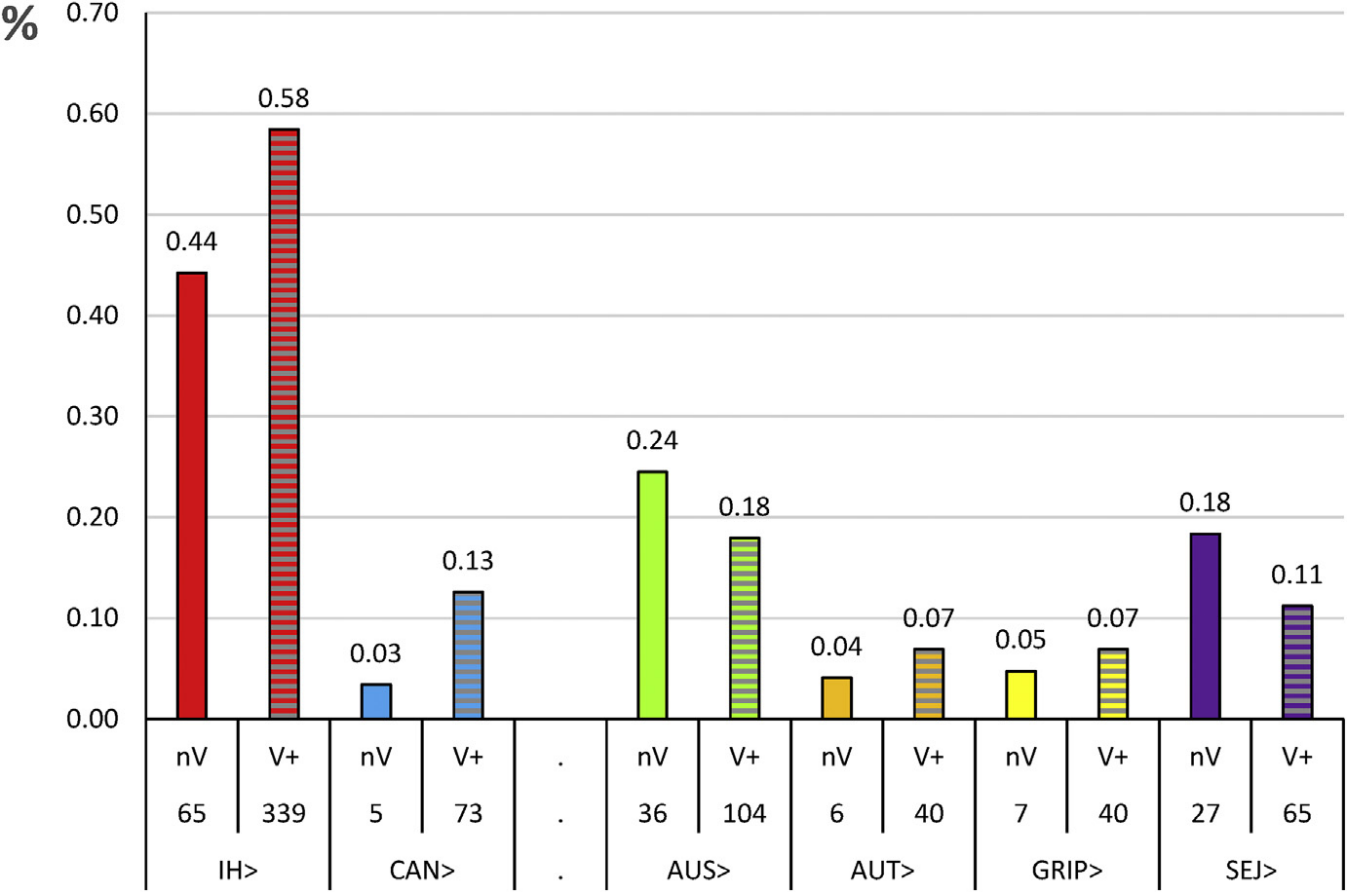
High titres (≥320) to each serogroup in percent of MAT positive dogs in naive (N = 147) and vaccinates (N = 580). nV: not vaccinated, V+: vaccinates, IH: Icterohaemorrhagiae, CAN: Canicola, AUS: Australis, AUT: Autumnalis, GRIP: Grippotyphosa, SEJ: Sejroë.

Conversely to the low titres, high titres against the other serogroups could not have been elicited by a previous vaccination and therefore expressed the incidence. For Australis, high titres were shown in 17.9% of cases (CI 95% = 15.1–20.7), followed by 11.2% for Sejroë (CI 95% = 9.1–13.8) and Grippotyphosa and Autumnalis with 6.9% (CI 95% = 4.8–8.4) and 6.9% (CI 95% = 4.5–8.1), respectively (Fig. 4).

### 3.3 Clinical results

Among the 147 leptospirosis cases of unvaccinated dogs, liver damage (L) was recorded in 85 dogs (57.8%, CI 95% = 49.8–67.8) and acute kidney injury (AKI) in 76 dogs (51.7%, CI 95% = 43.6–59.8). Both symptoms were recorded simultaneously in 14 dogs.

**Fig. 5:**
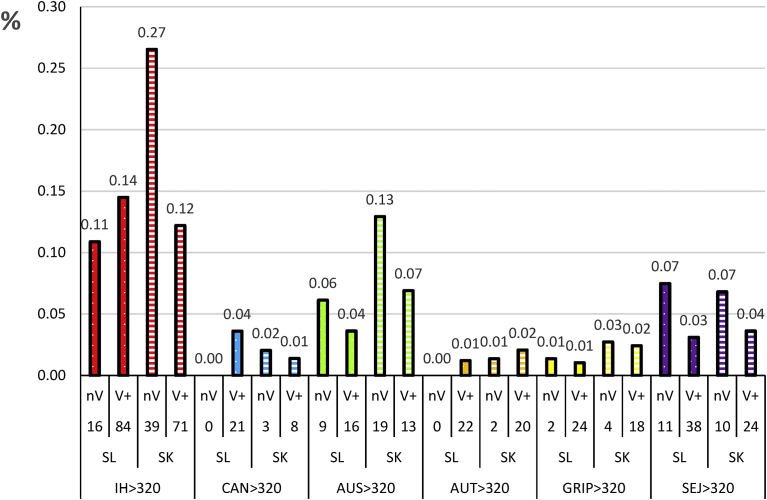
Serogroups related to severe hepatic and renal injuries in percent of number of dogs showing failures in naïve (nV) and vaccinate (V+) groups. IH: Icterohaemorrhagiae, CAN: Canicola, AUS: Australis, AUT: Autumnalis, GRIP: Grippotyphosa, SEJ: Sejroë, SL: severe liver injury, SK: severe kidney failure.

Severe hepatitis (42% of hepatic cases) was shown by 24.5% of the dogs (36/147). Severe kidney damage (90% of AKI cases) was shown by 46.9% of the 147 dogs. In naïve dogs, high titres against Icterohaemorrhagiae were related to 16 out of the 36 severe hepatitis cases, whereas high titres to Canicola were only related to 3 severe AKI cases. Whatever the other focused serogroup, high titres were mostly related to severe AKI cases. Other detected clinical aspects of leptospirosis were generally associated with one or two of these symptoms. The ratio of severe cases of liver injury and AKI was dependent from the serogroup (Fig. 5). All the serogroups showed a stronger renal effect, Sejroë excluded. Icterohaemorrhagiae-related renal cases of naïve dogs were significantly higher (P = 0.0019) than cases for the other serogroups.

Among the 580 confirmed cases in fully vaccinated dogs (V+), liver injury was recorded in 281 dogs (46.5%, CI 95% = 42.4–50.6) and AKI in 148 dogs (21.6%, CI 95% = 18.3–24.9). The remaining dogs exhibited a broad panel of symptoms, including fever, lameness, respiratory distress, gastroenteritis and bleeding.

The third grade of liver damage (54% among the hepatitis cases) was recorded in 151/580 dogs (26.03%, CI 95% = 22.5–29.6) and grades III to V kidney damage (78% among the AKI) were recorded in 116/580 dogs (20.00%, CI 95% = 16.7–23.3). Among the vaccinated dogs, 84 exhibiting the third grade of liver damage (14.5%) showed prominently high Icterohaemorrhagiae titres and 21 (3.6%) showed high Canicola titres. Icterohaemorrhagiae-related severe kidney damage was seen in 71 dogs (12.2%) and in 8 (1.4%) cases related to Canicola.

## 4 Discussion

Several limits of this study performed in privately owned pets exposed to field strains of pathogenic *Leptospira* species throughout France [17] depended of the selected criteria.

Clinical picture was the first selection filter. The clinical recording of cases in vaccinates and naïve dogs was implemented by the same practitioner population. To minimise recording bias, this study only focused on the easy to quantify biochemical parameters of the well-known issues among various aspects of polysystemic leptospirosis [18, 19, 20, 21, 22]. The biochemical levels were used to define cases and to perform statistical analyses. As MAT is very specific [6], sensitivity was improved by use of a low first sera dilution (1:20). Therefore, the final reciprocal titre consistent with the clinical picture was 320 (fourth dilution) instead of the widely used threshold of 400 [9, 23]. An infectious strain usually belongs to a single serovar belonging itself to a single serogroup. However, a serum commonly shows cross-reactivity for several serogroups. Therefore, MAT results are often considered as poor indicators of the serogroup to which belongs the infectious strain [24], but the highest titre against a given serogroup is statistically correlated to the serogroup inducing the last infection of a naive dog [25] whatever the prominent serovar reacting in MAT within this serogroup. Morozetti Blancoa [26] showed 79% agreement between the serogroup predicted by MAT and the isolate identification. Therefore, the highest titre was considered consistent to the infectious serogroup and lower titres as cross-reactions.

Our purpose was to estimate the global protection elicited by bacterins in vaccinates exposed to the most pathogenic serogroups encountered in France highlighted by MAT results of sick unvaccinated pets (Fig. 2). The MAT was performed and recorded in a single laboratory avoiding most laboratory bias [5, 16].

Sera provided by unvaccinated dogs gave positive results to a mean of 2.36 different serogroups, highlighting the high level of cross-reactivity within the 6 field serogroups. As expected vaccinated dog samples exhibited a higher mean of seroreactive serogroups (2.60). New antibodies raised towards the infectious strain are added to the post-vaccine agglutinating antibodies (Icterohaemorrhagiae and Canicola). They increased the cross-agglutinations [25]. In agreement with this hypothesis, if the Icterohaemorrhagiae and Canicola agglutinins were retrieved, cross-reactions dropped from 2.60 to 1.091 in the V+ dogs compared to 2.36 to 1.28 in the nV dogs.

Icterohaemorrhagiae remained the major infectious serogroup as shown by the highest incidence in naïve dogs (44.2%), whereas Canicola appeared as the least infectious with 3.4%. Similar lower exposure (approximately minus 40%) was confirmed in vaccinates even if the previous vaccination allowed serological result increase (Icterohaemorrhagiae incidence 58% and Canicola 12%). These results are in accordance with previous data [3, 7, 27, 28, 29, 30]. The numerous animal species able to shed Icterohaemorrhagiae compared to the restricted Canicola maintenance hosts could be an explanation of such different exposures of dogs.

Protection triggered by bacterins is claimed to be serogroup-specific [3, 7, 8]. The major liver failure decreased from 57.8% in naive dogs to 46.5% in vaccinates and AKI from 51.7% to 21.6% (P = 2.10^−5^). Moreover, the severe form of AKI (all serogroups included) significantly dropped in vaccinates from 46.9 to 20.0% (P = 6.10^−6^). The specificity of that protection was questionable in naturally exposed vaccinates [31, 32]. It was very noticeable that the rate of upper grades of AKI related to Icterohaemorrhagiae and Canicola serogroups significantly decreased from 60% (39/65) in naïve dogs to 20.9% (71/339) for Icterohaemorrhagiae and from 60% (3/5) to 10.9% (8/73) for Canicola. MAT high titres were the combination of the previous vaccination and the last infection and show that field infections boost the previous antibodies while the newly infected animals were clinically protected. Therefore, the potential consequences of this vaccination has to be explored for the 4 other heterologous serogroups (Australis, Autumnalis, Grippotyphosa and Sejroë).

It was hypothesised that natural exposure of vaccinates could not be higher than that of naïve dogs. However, high titres against Autumnalis and Grippotyphosa increased in vaccinated dogs compared to naïve dogs (Fig. 4). It has been suggested that both these serogroups serologically cross-react with Icterohaemorrhagiae and/or Canicola. In this case, similar antibody increase would be shown for high titres linked to severe liver or kidney damage. This was valuable for Autumnalis high titres, but not for Grippotyphosa (Fig. 5). Therefore, it was hypothesised that the major liver or kidney troubles taken into account in this study were not the prominent clinical signs in these Grippotyphosa cases. These results are in accordance with the proper name given to this serogroup (flu-like and typhus), suggesting a particular clinical impact of serovars belonging to this serogroup [2, 33].

Conversely, severe liver and kidney damage cases related to Australis and Sejroë high titres decreased (Fig. 5). Therefore, it can be assumed that the former Icterohaemorrhagiae and Canicola vaccination afforded an unexpected clinical protection against the major liver and kidney failure of leptospirosis cases induced by at least the 3 heterologous serogroups, Australis, Grippotyphosa and Sejroë.

It is well known that the widely cross-agglutinating saprophytic *L. biflexa* is unable to afford any protection [3, 10, 34], whereas cross-reactive in MAT, lipopolysaccharides (LPS) epitopes are unable to share this cross-protection, in accordance with the claimed specificity of the bacterins. Therefore, these results emphasise that the usual whole cell vaccines elicit a broader immune response [35]. Pathogenic *Leptospira spp* share many other common and specific antigens [36], such as lipL32/Hap1 and other outer membrane proteins [37, 38, 39, 40, 41]. A new field infection of a vaccinate would boost his previously acquired immunity against LPS epitopes and simultaneously against some of the specific outer membrane proteins [42, 43, 44]. During the host invading process, non-LPS-specific immunity may be enhanced, affording an early significant but not fully achieved and short-term cross-protection [34, 42]. In the complement of the reference MAT, newly developed enzyme-linked immunosorbent assays (ELISAs) using these various antigens for coating supported this immune response complexity [45, 46, 47]. In pets exposed to infectious field strains, specific LPS agglutinating antibodies are efficient but are only one of the factors affording the clinical protection against leptospirosis. The useful pattern of antigens to achieve a complete protection against pathogenic *Leptospira spp* remains to be identified [48].

In conclusion, whole cell bacterins afford a significant protection against field strains belonging to homologous serogroups as well as heterologous pathogenic *Leptospira spp* regardless of their composition.

## Declarations

### Author contribution statement

Geneviève André-Fontaine: Conceived and designed the experiments; Analyzed and interpreted the data; Wrote the paper.

Laetitia Triger: Performed the experiments; Analyzed and interpreted the data; Contributed reagents, materials, analysis tools or data.

### Funding statement

This research did not receive any specific grant from funding agencies in the public, commercial, or not-for-profit sectors.

### Competing interest statement

The authors declare the following conflict of interests: Genevieve Andre-Fontaine is an inventor holding patents related to leptospirosis. The founding sponsors had no role in the collection, analysis or data interpretation, in writing the manuscript or in the decision to publish the study. The other author declares no conflict of interest.

### Additional information

No additional information is available for this paper.

## References

[1] Goldstein R.E. (2010). Canine leptospirosis. Vet. Clin. North Am. Small Anim. Pract..

[2] Faine S., Adler B., Bolin, Perolat P. (1999). *Leptospira* and Leptospirosis.

[3] Adler B. (2015). Vaccines against leptospirosis: *Leptospira* and leptospirosis. Curr. Top. Microbiol. Immunol..

[4] Andre-Fontaine G. (2006). Canine leptospirosis – do we have a problem?. Vet. Mic..

[5] Andre-Fontaine G. (2016). Leptospirosis in domestic animals in France: serological results from 1988 to 2007. Rev. Sci. Tech. OIE.

[6] OIE (2014). Manual of Diagnostic Tests and Vaccines for Terrestrial Animals. http://www.oie.int/fileadmin/Home/eng/Health_standards/tahm/2.01.12_LEPTO.pdf.

[7] Ellis W.A. (2010). Control of change leptospirosis in Europe: time for a change?. Vet. Rec..

[8] Klaasen H.L.B.M., Adler B. (2015). Recent advances in canine leptospirosis: focus on vaccine development. Vet. Med. Res. Rep..

[9] Schuller S., Francey T., Hartmann K., Hugonnard M., Kohn B., Nally J.E. (2015). European consensus statement on leptospirosis in dogs and cats. J. Small Anim. Pract..

[10] Gautam R., Wu C.C., Guptill L.F., Potter A., Moore G.E. (2010). Detection of antibodies against *Leptospira* serovars via microscopic agglutination test in dogs in the United States, 2000–2007. J. Am. Vet. Med. Assoc..

[11] Sykes J., Hartmann K.F., Lunn G.E., Moore R.A.S., Goldstein R.E. (2010). ACVIM small animal consensus statement on leptospirosis: diagnosis, epidemiology, treatment, and prevention. J. Vet. Intern. Med..

[12] Kaneko J.J., Harvey H.W., Bruss M.L., Kaneko J.J., Harvey J.W., Bruss M.L. (1997). Clinical Biochemistry of Domestic Animals.

[13] Center S.A. (2007). Interpretation of liver enzymes. Vet. Clin. Small Anim..

[14] Anonymous: International Renal Interest Society: grading of acute kidney injury http://www.iris-kidney.com. (access dec 2013).

[15] Andre-Fontaine G. (2013). Diagnosis algorithm for leptospirosis in dogs: disease and vaccination effects on the serological results. Vet. Rec..

[16] Aviat F., Blanchard B., Michel V., Blanchet B., Branger C., Hars J. (2009). Leptospira exposure in the human environment in France: a survey in feral rodents and fresh water. Comp. Immunol. Microbiol. Infect. Dis..

[17] Scanziani E., Calcaterra S., Tagliabue S., Luini M., Giusti A.M., Tomba M. (1994). Serological findings in cases of acute leptospirosis in the dog. J. Small Anim. Pract..

[18] Sent U., Pothmann M. (1995). Atypical clinical course of an infection with L-interrogans Serovar saxkoebing in a dog. Kleintierpraxis.

[19] Nally J.E., Chantranuwat C., Wu X.Y., Fishbein M.C., Pereira M.M., Pereira Da Silva J.J. (2004). Alveolar septal deposition of immunoglobulin and complement parallels pulmonary hemorrhage in a Guinea pig model of severe pulmonary leptospirosis. Am. J. Pathol..

[20] Klopfleisch R., Kohn B., Plog S., Weingart C., Nöckler K., Mayer-Scholl A. (2010). An emerging pulmonary haemorrhagic syndrome in dogs: similar to the human leptospiral pulmonary haemorrhagic syndrome?. Vet. Med. Int..

[21] Tangeman L.E., Líttman M.P. (2013). Clinicopathologic and atypical features of naturally occurring leptospirosis ín dogs: 5l cases (2000–2010). J. Am. Vet. Med. Assoc..

[22] Miller M.D., Annis K.M., Lappin M.R., Lunn K.F. (2011). Variability in results of the microscopic agglutination test in dogs with clinical leptospirosis and dogs vaccinated against leptospirosis. J. Vet. Intern. Med..

[23] Craig S.B., Graham G.C., Burns M.-A., Dohnt M.F., Smythe L.D., Mckay D.B. (2009). A case of ‘original antigenic sin’ or just a paradoxical reaction in leptospirosis?. Ann. Trop. Med. Parasitol..

[24] Morozetti Blancoa R., Dos Santosa L.F., Galloway R.L., Caló Romero E. (2016). Is the microagglutination test (MAT) good for predicting the infecting serogroup for leptospirosis in Brazil?. Comp. Immunol. Microbiol. Infect. Dis..

[25] Houvers D.J., Goris M.G., Abdoel T., Kas J.A., Knobbe S.S., Van Dongen A.M. (2011). Agglutinating antibodies against pathogenic *Leptospira* in healthy dogs and horses indicate common exposure and regular occurrence of subclinical infections. Vet. Mic..

[26] Nardone A., Campese C., Postic D., Andre-Fontaine G., Lienard M., Baranton G. (2001). Les facteurs de risque de leptospirose en France : une étude cas-témoins nationale (1999). Med. Maladies Infect..

[27] Barmettler R., Schweighauser A., Bigler S., Grooters A.M., Francey T. (2011). Assessment of exposure to Leptospira serovars in veterinary staff and dog owners in contact with infected dogs. J. Am. Vet. Med. Assoc..

[28] Raghavan R., Brenner K., Higgins J., Van Der Merwe D., Harkin K.R. (2011). Evaluations of land cover risk factors for canine leptospirosis: 94 cases (2002–2009). Prev. Vet. Med..

[29] Harland A., Cave N., Jones B., Benschop J., Donald J.J., Midwinter A.C. (2013). A serological survey of leptospiral antibodies in dogs in New Zealand. J. N. Z. Vet. J..

[30] Arent Z.J., Andrews S., Adamama-Moraitou K., Gilmore C., Pardali D., Ellis W.A. (2012). Emergence of novel Leptospira serovars: a need for adjusting vaccination policies for dogs?. Epidemiol. Infect..

[31] Klaasen H.L.B.M., Van Der Veen M., Molkenboer M.J.C.H., Sutton D. (2013). A novel tetravalent Leptospira bacterin protects against infection and shedding following challenge in dogs. Vet. Rec..

[32] Rosario L.A., Arencibia D.F., Suarez Y.E., Infante J.F., Valdés B.Y., Batista N. (2012). Cross-protection among unrelated leptospira pathogens serovars: an unfinished story. Adv. Clin. Exp. Med..

[33] Kmety E., Dikken H. (1993). Classification of the Species *Leptospira interrogans* and History of its Serovars.

[34] Srikram A., Zhang K., Bartpho T., Lo M., Hoke D.E., Sermswan R.W. (2011). Cross-protective immunity against leptospirosis elicited by a live, attenuated lipopolysaccharide mutant. J. Infect. Dis..

[35] Gitton X., Buggin Daubie M., Andre F., Ganiere J.P., Andre-Fontaine G. (1994). Recognition of *Leptospira interrogans* antigens by vaccinated or infected dogs. Vet. Mic..

[36] Haake D.A., Matsunaga J. (2010). *Leptospira:* a spirochaete with a hybrid outer membrane. Mol. Microbiol..

[37] Cullen P.A., Cordwell S.J., Bulach D., Haake D.A., Adler B. (2002). Global analysis of outer membrane proteins from *Leptospira interrogans* serovar Lai. Infect. Immun..

[38] Pinne M., Haake D.A. (2013). LipL32 is a subsurface lipoprotein of *Leptospira interrogans*: presentation of new data and reevaluation of previous studies. PLoS One.

[39] Thomé S., Lessa-Aquino C., Ko A.I., Lilenbaum W., Medeiros M.A. (2014). Identification of immunodominant antigens in canine leptospirosis by Multi-Antigen Print ImmunoAssay (MAPIA). BMC Vet. Res..

[40] Teixeira A.F., De Morais Z.M., Kirchgatter K., Calo Romero E., Vasconcellos S.A., Nascimento A.L.T.O. (2015). Features of two new proteins with OmpALike domains identified in the genome sequences of Leptospira interrogans. PLoS One.

[41] Branger C., Chatrenet B., Gauvrit A., Aviat F., Aubert A., Bach J.M. (2005). Protection against *Leptospira interrogans* challenge by DNA immunization with Hemolysin Associated Protein 1 (Hap1) encoding gene in gerbils. Infect. Immun..

[42] Felix S.R., Hartwig D.D., Argondizzo A.P.C., Silva E.F., Seixas F.K., Amilton C. (2011). Subunit approach to evaluation of the immune protective potential of leptospiral antigens. Clin. Vacc. Immunol..

[43] Corsi D.I.C., Paldes Gonçales A., De Morais Z.M., De Souza G.O., Miraglia F., Abreu P.A.E. (2014). Cross-protection between experimental anti-leptospirosis bacterins. Braz. J. Microbiol..

[44] Sonrier C., Branger C., Michel V., Ruvoen-Clouet N., Ganiere J.P., Andre-Fontaine G. (2000). Evidence of cross protection within *Leptospira interrogans ss* in an experimental model. Vaccine.

[45] Dey S., Mohan C.M., Kumar T.M., Ramadass P., Nainar A.M., Nachimuthu K. (2004). Recombinant LipL32 antigen-based single serum dilution ELISA for detection of canine leptospirosis. Vet. Microbiol..

[46] Ye C., Yan W., Xiang H., He H., Yang M., Ijaz M. (2014). Recombinant antigens rLipL21, rLoa22, rLipL32 and rLigACon4-8 for serological diagnosis of leptospirosis by enzyme-linked immunosorbent assays in dogs. PLoS One.

[47] Andre-Fontaine G., Aviat F., Marie J.L. (2015). Undiagnosed leptospirosis cases in naïve and vaccinated dogs: properties of a serological test based on a synthetic peptide derived from Hap1/LipL32 (residues 154-178). Comp. Immunol. Microbiol. Infect. Dis..

[48] Dellagostin O., Grassmann A.A., Rizzi C., Schuch R.A., Jorge S., Oliveira T.L. (2017). Reverse vaccinology: an approach for identifying leptospiral vaccine candidates. Int. J. Mol. Sci..

