# Erratum to “MAT cross-reactions or vaccine cross-protection: retrospective study of 863 leptospirosis canine cases” [Heliyon 4 (11) (November 2018) e00869]

**DOI:** 10.1016/j.heliyon.2018.e01027

**Published:** 2018-12-17

**Authors:** Geneviève André-Fontaine, Laetitia Triger

**Affiliations:** Laboratoire de Bactériologie Médicale et Moléculaire des Leptospires, École Nationale Vétérinaire, ONIRIS, Route de Gachet, CS 40706, 44307 Nantes Cedex 03, France

In the original published version of this article, the publisher accidentally inverted figure 2 and figure 3 including the figure legends. The figure and figure legend published as ‘Fig. 2’ should instead have been published as ‘Fig. 3’ and vice versa. The publisher apologises for the error. Both the HTML and PDF versions of the article have been updated to correct the error.

